# Regulatory challenges and global trade implications of genome editing in agriculture

**DOI:** 10.3389/fbioe.2025.1609110

**Published:** 2025-06-19

**Authors:** Danilo Fernández Ríos, Silverio Andrés Quintana, Pilar Gómez Paniagua, Andrea Alejandra Arrúa, Gustavo René Brozón, Moises Santiago Bertoni Hicar, Andrés Castro Alegría, María Florencia Goberna

**Affiliations:** ^1^ Facultad de Ciencias Exactas y Naturales, Universidad Nacional de Asunción, San Lorenzo, Paraguay; ^2^ Doctorado en Ciencias Agrarias, Universidad San Carlos, Asunción, Paraguay; ^3^ Grupo de Investigación Mycology Investigation and Safety Team, Centro Multidisciplinario de Investigaciones Tecnológicas, Universidad Nacional de Asunción, San Lorenzo, Paraguay; ^4^ Centro Multidisciplinario de Investigaciones Tecnológicas, Universidad Nacional de Asunción, San Lorenzo, Paraguay; ^5^ Unión de Gremios de la Producción, Asunción, Paraguay; ^6^ Coordination of Innovation and Biotechnology, National Bioeconomy Directorate, Sub-secretariat of Agricultural Production and Forestry, Secretariat of Agriculture, Livestock and Fisheries, Buenos Aires, Buenos Aires, Argentina

**Keywords:** genome editing, new breeding techniques, regulatory science, international trade, harmonization

## Abstract

Genome editing revolutionized agriculture by improving crop productivity, disease resistance, and adaptation to adverse climatic conditions. However, it has faced significant regulatory challenges due to divergent regulations between regions. Although Europe classified these organisms as genetically modified organisms, Africa, Asia, and Latin America implemented more flexible regulatory frameworks, which encouraged innovation and the participation of small companies. These differences could generate high costs, delays in commercialization, and difficulties in product traceability, affecting research and development decisions. This article analyzes the main regulatory challenges and their impact on global trade, proposing strategies for regulatory harmonization to promote transparency, reduce trade barriers, and maximize the potential of these technologies in the face of global challenges such as food security and climate change.

## 1 Introduction

Genome editing technologies^1^ have advanced significantly in recent years, expanding their applications in agriculture. These tools allow precise changes to the genetic characteristics of crops, favoring improvements in productivity, disease resistance, and adaptability to changing climatic conditions (Rajput et al., 2021; Zenda et al., 2021; Das et al., 2022; Ntsomboh-Ntsefong et al., 2023; Groover et al., 2024).

However, their adoption faces significant regulatory challenges due to the diversity of existing policies at the global level. The regulations governing genome editing vary considerably among regions, which generates uncertainty and complexity for its implementation in international trade and agriculture (Tachikawa and Matsuo, 2023; Rosado, 2024).

In this article, we comment on the regulatory and trade challenges arising from these policy discrepancies, highlighting their implications and proposing strategies to promote greater global regulatory harmonization.

## 2 Regulatory landscape and challenges

The distinction between process- and product-based regulations represents a central axis in the governance of genome editing. In a process-based regulatory system, oversight is typically triggered by the use of recombinant DNA technology, rather than by the properties of the resulting organism. This approach originated in the early 1990s with a regulatory framework that distinguished conventional breeding methods (such as hybridization and mutagenesis) from genetic engineering involving the insertion of DNA (EUR-Lex, 1990). The term “genetically modified organism” (GMO) emerged to capture this technical boundary.

In contrast, product-based regulatory systems assess organisms based on the characteristics of the final product, regardless of the method used to generate them. Canada’s regulatory model for “plants with novel traits” exemplifies this approach (Sprink et al., 2016). According to the Canadian Food Inspection Agency, a novel trait is defined as one that is new to the local environment and has the potential to affect a plant’s safety for human health or the environment, regardless of whether it was introduced through genome editing, conventional breeding, or mutagenesis (Government of Canada, 1990; Canadian Food Inspection Agency, 2020).

This regulatory dichotomy has prompted scientific institutions to advocate for product-based, evidence-driven governance. The European Academies’ Science Advisory Council concluded in 2013 that genetic engineering does not pose intrinsically greater risks than conventional breeding and advocated for a regulatory shift based on product traits rather than the methods (European Academies Science Advisory Council and Deutsche Akademie der Naturforscher Leopoldina, 2013). This view is supported by decades of empirical research showing that risk is associated with the function and expression of novel traits and not the mechanism of their introduction (Heap, 2013; Hartung and Schiemann, 2014; Sprink et al., 2016). In nature, similar genetic alterations occur spontaneously through mutations, recombination, or horizontal gene transfer, challenging the rationale for process-based oversight (Fernández Ríos et al., 2025). From a biosafety perspective, risk estimates for some products obtained through genome editing should thus align with those for naturally occurring genetic variation or conventionally bred plants (Hernández-Soto and Gatica-Arias, 2024).

Moreover, the enforcement of process-based regulations becomes technically unworkable when it cannot be determined whether a product was generated using a specific technique. For example, if a mutation produced by CRISPR/Cas cannot be distinguished from that arising through mutagenesis, then the ability to ensure compliance and implement policies for unapproved GMOs in seeds becomes functionally impossible. This outcome undermines the regulatory goals of traceability and safety assurance (Sprink et al., 2016). Although some scholars have argued against framing the debate as a binary opposition between process and product regulation (Kuzma, 2016) and call for more integrative approaches, it remains essential to recognize that product characteristics must ultimately form the basis for regulatory coherence and proportionality (McHughen, 2016).

**FIGURE 1:**
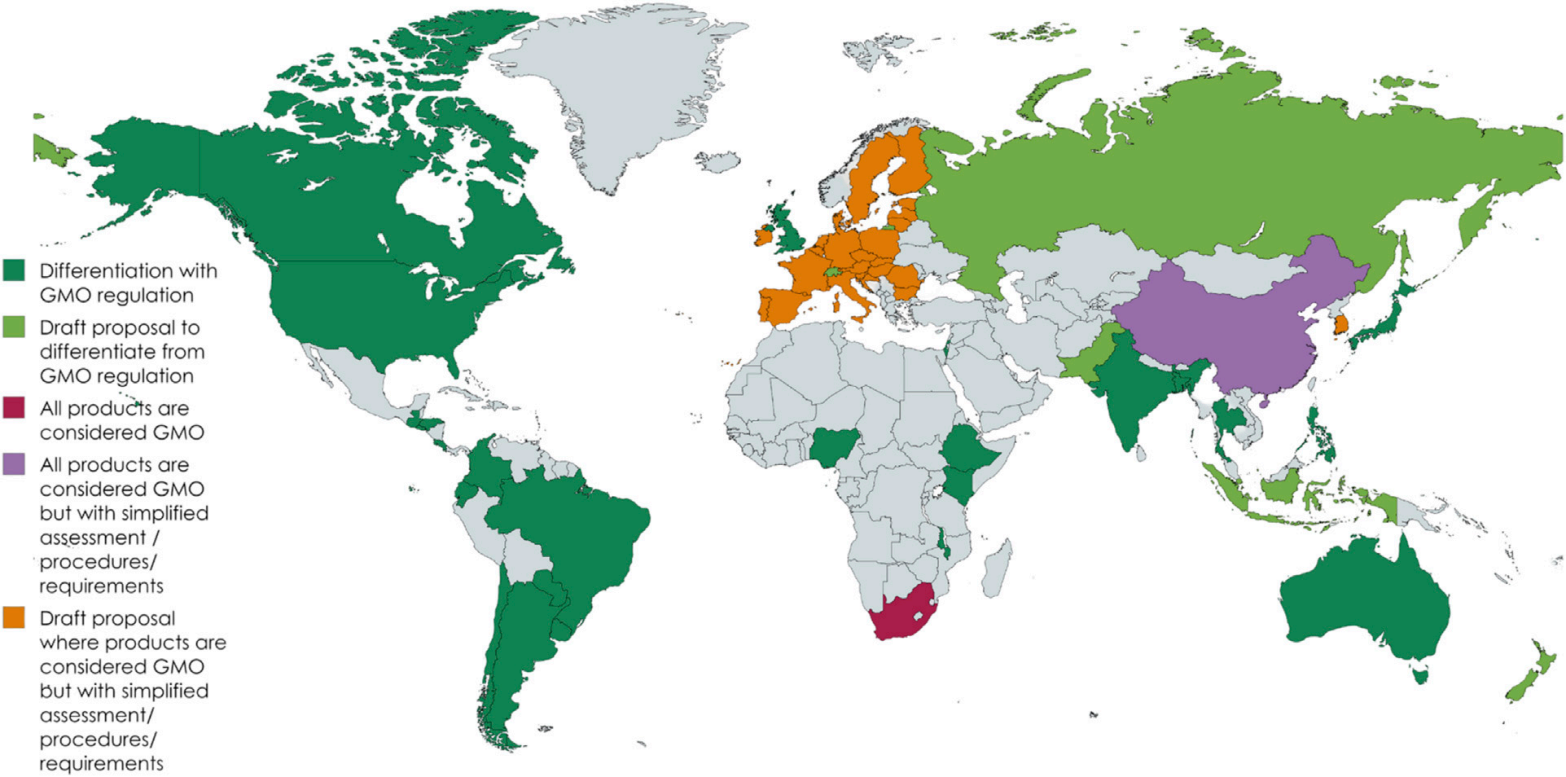
Global genome editing policy development.

Genome editing regulations vary considerably among regions (Figure 1), such as the European Union, Africa, Asia, and Latin America (Zarate et al., 2023; Sprink and Wilhelm, 2024). In the European Union, genome-edited organisms are currently classified as GMOs, although proposals to categorize certain edited products with a limited and predefined number of genetic changes in a differentiated manner are being evaluated (Ahmad et al., 2023; Purnhagen et al., 2023). Although pre-marketing requirements are not yet fully defined, they are likely to include measures such as labeling, segregation, and specific regulations for handling. Post-marketing requirements, such as additional monitoring, are also under discussion and may include more detailed regulations in the future.

However, more flexible regulatory approaches have been adopted in Asian countries, such as China and India. Since 2022, China has implemented regulations that shorten the approval times for products derived from new breeding techniques (NBTs) to 1–2 years. This framework prioritizes food safety and environmental impact assessments. Pre-market requirements include assessment processes similar to those applied to GMOs, whereas post-market provisions mandate labeling to ensure transparency and consumer awareness in the marketplace (USDA, 2023). Meanwhile, India has adopted a similarly flexible regulatory approach, excluding products developed through SDN1 (deletions or substitutions without adding foreign DNA) and SDN2 (using an exogenous DNA template but not integrating foreign DNA into the final genome) from being classified as GMOs, provided they do not contain foreign DNA. These products are exempt from biosafety assessments, and their status is certified by an Institutional Biosafety Committee, allowing them to be treated as conventional crops (Ministry of Environment, Forest, and Climate Change, 2022; Groover et al., 2024). This approach fosters innovation by reducing development costs and time and accelerates the commercialization of genome-edited products. India thus seeks to promote technological advances in agriculture (FAO, 2022).

On the other hand, in Africa, Burkina Faso, Ethiopia, Kenya, Nigeria, and Malawi are advancing toward adaptive regulatory frameworks for genome editing based on the principles of case-by-case review and risk proportionality. Kenya and Nigeria have developed guidelines that distinguish between conventional, intermediate, and transgenic products, applying different levels of regulation depending on the nature of genetic modification (Adegbaju et al., 2024; Groover et al., 2024). Both systems include early consultation mechanisms to determine the appropriate regulatory pathway, thereby providing greater clarity and predictability for developers. Ethiopia has drafted regulations excluding certain genome-edited products without foreign DNA, with proposals currently under review (Groover et al., 2024). This growing trend positions Africa as an emerging reference point for the development of regulatory frameworks that combine scientific rigor with flexibility to facilitate responsible innovation (Rabuma et al., 2024; Akinbo et al., 2025).

On the other hand, regulations in some countries in Latin America establish prior consultation on whether a product derived from NBTs will be considered conventional or not, providing clarity and predictability from the early stages of development (Fernández Ríos et al., 2024; Hernández-Soto and Gatica-Arias, 2024; Pérez et al., 2024; Sánchez, 2024; Brant et al., 2025). If the final product does not contain foreign DNA or introduce a novel genetic combination—and could have been generated through natural processes—it is classified as a conventional product, which significantly reduces regulatory costs and opens up opportunities for small and medium-sized companies to participate. This framework also encourages the generation of more productive varieties adapted to market demands, boosting agricultural innovation and regional competitiveness (Lubieniechi et al., 2025).

Regulatory differences create barriers to the adoption of genome editing technologies, affecting the competitiveness and international trade of agricultural products. Table 1 presents a comparative summary of regulatory approaches in different regions.

**TABLE 1:** Comparative overview of genome editing policies across countries and regions.

Region/Country	Regulatory approach	Reference
Argentina, Brazil, Chile, Colombia, Costa Rica, El Salvador, Guatemala, Honduras, Paraguay, Ecuador, and Uruguay	Case-by-case assessment of products obtained through genome editing. If the final product does not have a new combination of genetic material, it is considered conventional	Fernandes et al. (2024), Fernández Ríos et al. (2024), Goberna et al. (2024), Hernández-Soto and Gatica-Arias (2024), Sánchez (2024)
Australia	Has revised its regulations to exclude SDN1 from oversight. Modifications without the introduction of foreign DNA not interpreted as additional risks	Thygesen (2019), Jones et al. (2024)
Bangladesh	Case-by-case approach. Products obtained through SDN1/SDN2, with no foreign DNA, excluded from strict regulations	Groover et al. (2024)
Burkina Faso, Ghana, Kenya, Malawi, and Nigeria	Guidelines using a case-by-case approach, excluding certain genome-edited products without foreign DNA from strict regulations	Adegbaju et al. (2024), Groover et al. (2024)
Canada	Applies product-based approach. Assesses final traits of the organism, not the technique used to develop it. Plants without foreign DNA are exempt from strict regulations^a^	Lassoued et al. (2024)
China	Regulation prioritizes food safety and environmental risk assessment. Pre-market requirements include risk assessment processes similar to those applied to GMOs, while post-market provisions provide mandatory labeling, thus ensuring transparency and traceability of products on the market	USDA (2023)
Ethiopia	Drafted guidelines exclude certain NBT products from strict regulations, still under the process of review and approval	Groover et al. (2024)
European Union	Genome-edited organisms considered GMOs. There are proposals to categorize certain edited products, with a limited and pre-defined number of genetic changes in a differentiated manner. Pre-market requirements not yet fully defined, likely to include labeling, segregation, and specific provisions for handling	Purnhagen et al. (2023), Molitorisová et al. (2024)
India	Products obtained through SDN1/SDN2, with no foreign DNA, not considered GMO	Ministry of Environment Forest and Climate Change (2022), Groover et al. (2024)
Indonesia and Vietnam	A draft has been proposed to exempt certain genome-edited products from strict regulations. Still under discussion, awaits implementation	Yang and Zhou (2024)
Japan	Case-by-case approach, excluding certain genome-edited products that do not contain foreign DNA from strict regulations	Tomita (2024)
Philippines and Singapore	Case-by-case approach, excluding products without foreign DNA from strict regulations	Groover et al. (2024)
Russia	It implemented a decision for a research and development program that classifies genome-edited products as similar to conventional products	Dobrovidova (2019)
South Africa	Considers NBT, including genome editing, such as GMOs	Berger, 2022; ACB (2024)
South Korea	Currently updating regulatory frameworks for NBTs. Currently, these techniques are regulated under the law on Living Modified Organisms (LMOs)	Yang and Zhou (2024)
Thailand	Exempts products obtained through SDN1 from strict regulations; for SDN2 and SDN3, without foreign DNA, assessment performed case by case to determine applicable regulation	Groover et al. (2024)
United Kingdom	Measures were implemented to allow field trials of genome-edited plants, requiring only one registration	Groover et al. (2024)
United States	Case-by-case approach. The Department of Agriculture and Environmental Protection Agency assesses products according to their competencies; the Food and Drug Administration offers voluntary consultations and does not require mandatory prior review	Hoffman (2021), Groover et al. (2024)

^a^ According to current genome editing regulations, any crop variety with herbicide tolerance will invariably be classified as a plant with a novel trait (Lubieniechi et al., 2025).

## 3 Trade barriers and opportunities in genome editing

Regulatory discrepancies between regions affect the global trade of genome-edited products by increasing costs, delaying approvals, and reducing market access. Developers must navigate diverse regulatory frameworks, requiring adaptation to local rules and often additional testing, documentation, and procedures that vary by country. These challenges not only slow commercialization but also increase costs, limiting companies’ ability to bring innovations to market efficiently. Small and medium-sized developers are particularly affected as they have fewer resources to meet multiple regulatory requirements and face greater barriers to entry (Kalaitzandonakes et al., 2023). In addition, regulatory uncertainty discourages investment in R&D as companies tend to prioritize crops with a lower risk of facing trade barriers (Lassoued et al., 2018). This could limit the potential of genome editing to address global issues such as food security and climate change.

On the other hand, variability in pre- and post-market requirements between regions raises concerns about transparency in the use of genome editing technologies. These disparities reduce the availability of information to consumers and complicate risk management in the global trade of agricultural products (Brinegar et al., 2017).

To address these challenges, experts recommend advancing regulatory harmonization mechanisms, drawing inspiration from successful models in countries where regulation focuses on the final product (May et al., 2022; Lassoued et al., 2024). Additionally, establishing bilateral and multilateral agreements could help align regulatory criteria and promote convergence.

## 4 Discussion

The global regulatory landscape for genome editing in agriculture is characterized by significant heterogeneity, ranging from strict process-based systems to more flexible product-based approaches. This diversity creates complex and often significant barriers to the advancement and adoption of genome editing technologies.

One primary barrier to innovation and competitiveness is the adoption of strict regulations in which all genome-edited organisms are classified as GMOs. This approach subjects genome-edited crops to the same approval processes as GMOs, regardless of whether foreign DNA is present in the final product or whether the genetic change could have occurred naturally or through conventional breeding. Such overly burdensome regulations increase the cost of bringing products to the market, reduce the returns on investment, and create investment uncertainty, which discourages innovation, especially from smaller developers and public research institutions. The time and resources required to navigate these complex regulatory pathways can divert efforts from R&D.

In contrast, regulatory frameworks adopted by some Latin American countries tend to be more innovation-friendly. When no foreign DNA is present in the genome-edited product and a change could have arisen through conventional breeding, these countries often exempt such products from GMO regulations. This streamlines the path to the market, provides greater regulatory certainty for developers, and encourages investment by reducing the likelihood of costly and time-consuming regulatory delays. Argentina’s prior consultation instances (PCIs) exemplify how such frameworks can successfully facilitate agricultural innovation (Goberna et al., 2022; Goberna et al., 2024).

However, even with more flexible frameworks in some regions, the lack of international harmonization remains a significant obstacle. Differing regulatory requirements across countries can disrupt international trade, increase compliance costs, and delay the commercialization of new technologies, especially for smaller developers who must navigate a patchwork of regulations.

A lack of transparency, predictability, or a clear scientific basis in regulatory processes increases the risk for innovators, often discouraging investment in genome editing. Developers require science-based, transparent, and risk-proportionate regulations to invest confidently and bring genome-edited products to market.

Although genome editing holds great potential to address the United Nations Sustainable Development Goals, such as Zero Hunger, Good Health and Well-Being, Climate Action, and Life on Land, disjointed and inadequate regulatory frameworks can pose major challenges to biotechnological innovation (Jenkins et al., 2021; Robusti and Farina, 2025). Excessively strict process-based regulations, lack of international alignment, and regulatory uncertainty all contribute to higher costs, development delays, and reduced incentives for the adoption of genome-edited crops.

Concrete recommendations for regulatory convergence are urgently needed, given the limited number of genome-edited products currently available in the market. This early stage presents an opportunity to align frameworks before broader commercialization takes place. To strengthen the coherence and efficiency of genome editing oversight, we propose recommendations that regulatory authorities and harmonization initiatives can adopt.

The comparators used in regulatory evaluations should shift from the traditional focus on GMOs to those based on conventionally bred products (Hernández-Soto and Gatica-Arias, 2024). This adjustment would enable a risk-proportionate approach by aligning regulatory scrutiny with the characteristics of the final product rather than the method of genetic modification, thereby acknowledging the biological equivalence between certain genome-edited outcomes and those obtained using conventional techniques.

Administrative resolutions should explicitly classify genome-edited organisms as conventional when they do not contain foreign DNA or novel genetic combinations. This formal legal qualification enhances clarity across related regulatory procedures, including seed registration, labeling, and commercial authorization, while ensuring consistency with national and international biosafety frameworks.

Molecular characterization requirements should be limited to the species level when the edited trait falls within the range of natural or induced variation. Requiring varietal-level analyses in such instances imposes an unnecessary technical burden and risks regulatory disproportionality. A species-level focus provides sufficient resolution for compliance verification without impeding product development timelines.

Regulatory frameworks should incorporate formal recognition of prior determinations made by competent authorities in countries with compatible biosafety systems (Hernández-Soto and Gatica-Arias, 2024). Such decisions can serve as valid references for expedited assessments, facilitating regulatory convergence, improving efficiency, and reinforcing trust among jurisdictions without necessitating redundant evaluations.

A recent example of regulatory cooperation is the *Agências de Biossegurança em Rede para Biotecnologia* (ABRE-Bio) Memorandum of Understanding between Argentina and Brazil, which establishes institutional coordination between regulatory agencies to synchronize the evaluation and approval of agricultural biotechnology products (MECON and MCTI, 2022). This initiative aims to minimize regulatory asynchronies that could disrupt trade while ensuring safety for agroecosystems and food security at both national and regional levels. Benefits of this system include feasibility pre-assessment services for small and medium-sized developers without legal representation in destination markets, joint determination of the regulatory status of NBT-derived products, and significant reductions in regulatory timelines for all users (Secretaría de Agricultura, Ganadería y Pesca, 2023). Recently, Paraguay and Uruguay signed the agreement, and ABRE-Bio is open to any country interested in joining (Astarita et al., 2025; Lewi et al., 2025).

Similarly, in Australia and New Zealand, a joint food regulation system managed by Food Standards Australia New Zealand (FSANZ) ensures that genetically modified foods, including those developed using genome editing, are assessed and approved under unified safety criteria before commercialization (FSANZ, 2025). This model offers a regional example of coordinated oversight that reduces trade barriers while safeguarding consumer health.

The New Partnership for Africa’s Development (NEPAD) program represents a significant strengthening of national regulatory capacities for both GMOs and genome-edited products (AUDA-NEPAD, 2018; Rabuma et al., 2024). NEPAD has actively promoted regional harmonization of biosafety policies, fostering cooperation among Member States and integrating socio-economic assessments alongside environmental considerations as part of regulatory decision-making (Adegbaju et al., 2024). This approach positions the region as an emerging leader ready to adopt new agricultural technologies.

Finally, genome editing oversight should be grounded in a precise legal definition that invokes conventional breeding. Clarifying this legal boundary would enable more predictable decision-making, lower compliance costs, and promote equitable access to innovation across both the public and private sectors.

## Data Availability

The original contributions presented in the study are included in the article/Supplementary Material; further inquiries can be directed to the corresponding authors.

## Footnotes

1 Refers to novel techniques for manipulating the genome with greater precision than that of pre-existing genetic engineering methods. These technologies have major implications for innovation across biomedicine, agriculture, and industrial biotechnology, owing to their more exact, less expensive, and easier genetic manipulation (Shukla-Jones et al., 2018).
